# Recent advances in transesterification for sustainable biodiesel production, challenges, and prospects: a comprehensive review

**DOI:** 10.1007/s11356-024-32027-4

**Published:** 2024-01-23

**Authors:** Sabah Mohamed Farouk, Aghareed M. Tayeb, Shereen M. S. Abdel-Hamid, Randa M. Osman

**Affiliations:** 1 Chemical Engineering Department, Egyptian Academy for Engineering and Advanced Technology (EA&EAT), affiliated to the Ministry of Military Production, Km. 3 Cairo Belbeis Desert Rd., Cairo Governorate, 3066 Egypt; 2 Faculty of Engineering, Minia University, Misr Aswan Agricultural Rd., EL MAHATTA, Menia Governorate, 2431384 Egypt; 3 Chemical Engineering and Pilot Plant Department, National Research Centre (NRC), 33 El Bohouth St., Dokki, 12622 Giza Governorate Egypt

**Keywords:** Sustainable biodiesel, Homogeneous catalysis, Heterogeneous catalysis, Nanocatalysts, Transesterification, Kinetics, Characterization, Challenges and prospect

## Abstract

Biodiesel, a renewable and sustainable alternative to fossil fuels, has garnered significant attention as a potential solution to the growing energy crisis and environmental concerns. The review commences with a thorough examination of feedstock selection and preparation, emphasizing the critical role of feedstock quality in ensuring optimal biodiesel production efficiency and quality. Next, it delves into the advancements in biodiesel applications, highlighting its versatility and potential to reduce greenhouse gas emissions and dependence on fossil fuels. The heart of the review focuses on transesterification, the key process in biodiesel production. It provides an in-depth analysis of various catalysts, including homogeneous, heterogeneous, enzyme-based, and nanomaterial catalysts, exploring their distinct characteristics and behavior during transesterification. The review also sheds light on the transesterification reaction mechanism and kinetics, emphasizing the importance of kinetic modeling in process optimization. Recent developments in biodiesel production, including feedstock selection, process optimization, and sustainability, are discussed, along with the challenges related to engine performance, emissions, and compatibility that hinder wider biodiesel adoption. The review concludes by emphasizing the need for ongoing research, development, and collaboration among academia, industry, and policymakers to address the challenges and pursue further research in biodiesel production. It outlines specific recommendations for future research, paving the way for the widespread adoption of biodiesel as a renewable energy source and fostering a cleaner and more sustainable future.

## Introduction

Fossil fuels are responsible for most of the world’s energy needs, endangering the environment. The consumption of fossil fuel products like coal and petroleum has increased because of ongoing globalization and industrialization (Touqeer et al. 2020). Around 580 TJ of energy is needed annually for the entire planet, and a staggering 80% of that energy is supplied by burning traditional fossil fuels. Greater energy use is required by expanding the population and improving accessibility, which increases the depletion of fossil fuel reserves (Martchamadol and Kumar 2012). Finding an alternate energy source, like biodiesel, is essential for improving energy security for economic development (Oh et al. 2002; Um and Kim 2009). Biodiesel is a renewable, sustainable, and biodegradable fuel with low greenhouse gas emissions (Sharma and Singh 2009; Lee et al. 2011). Oils from plants and animals, as well as other lipids such as triacylglycerides (TAGs), can be converted into biodiesel (Hoekman and Robbins 2012). The process of making biodiesel, called transesterification or alcoholysis, typically requires the use of catalysts such as acids, bases, and enzymes (Ong et al. 2014). Catalysts can exist in either a homogeneous or heterogeneous phase. A homogeneous catalyst undergoes alcoholysis in the same phase, often liquid, as the reactants. In contrast, a heterogeneous catalyst is in a different phase, often one that is not liquid, from the reactants (Ruhul et al. 2015). Nanomaterials have become increasingly important in enhancing the production of biodiesel. The weight of the catalyst, the reaction temperature, and the oil-to-alcohol ratio are all reduced when a nanocatalyst is used (Dhawane et al. 2018; Ghosh and Halder 2022). It has already been proven that nanocatalysts play a key role in increasing response velocity. Additionally, it significantly lowers the reactants’ activation energy. This study addresses the need to keep up with the rapidly changing field of biodiesel production. By conducting thorough surveys and reviews, researchers can stay informed about the latest advancements and challenges. This approach helps focus research efforts by identifying promising methodologies and comparing their performance. The production of biodiesel encounters several challenges, including costly feedstocks, environmental concerns, and the quest for improved efficiency. Through a meticulous review of relevant literature, researchers can identify these challenges and propose potential solutions. This review serves as a valuable resource, synthesizing information from different sources to provide a comprehensive understanding of the current state of biodiesel production technology. Policymakers and industry leaders rely on up-to-date insights to make informed decisions that shape the future of the biodiesel industry. Concise surveys and reviews effectively communicate the latest information, enabling informed decision-making. Additionally, this review promotes collaboration and knowledge sharing within the biodiesel research community, facilitating the development of new and better technologies. It also educates the public about biodiesel production and its potential benefits, potentially increasing support and encouraging the adoption of biodiesel as a sustainable alternative to petroleum-based fuels. Literary, and in detail, this paper offers a comprehensive review of biodiesel production, encompassing key topics such as feedstock selection, the transesterification process, the recovery and reusability of nanoparticles, the benefits and challenges of biodiesel use in engines, and the significance of techno-economic analysis. It begins with various feedstocks, including used oils, animal fats, algae, and edible and inedible vegetable oils, which are chosen for their potential to produce biodiesel. The review then highlights biodiesel as a promising alternative to fossil fuels, exploring its environmental benefits, renewability, and potential to reduce dependence on fossil fuels. The transesterification process, a crucial step in biodiesel production, is discussed in detail, covering reaction mechanisms, catalysts, influencing factors, and challenges associated with optimization. Additionally, various methods and strategies, including solid supports, magnetic separation, centrifugation, and surface modification, have been explored for the recovery and reusability of nanoparticles as catalysts in biodiesel production. These approaches offer promising prospects for enhancing the efficiency and sustainability of biodiesel synthesis while reducing costs and environmental impacts. The benefits of using biodiesel in engines, including improved lubricity, reduced emissions, and potential performance enhancements, are discussed. Lastly, this review emphasizes the importance of techno-economic analysis in assessing the financial viability and sustainability of biodiesel production, considering factors such as capital investment, operating costs, feedstock prices, and governmental policies. Overall, this comprehensive review serves as a valuable resource for researchers, engineers, and policymakers in the biodiesel industry.

## Feedstocks for biodiesel production

**Fig. 1:**
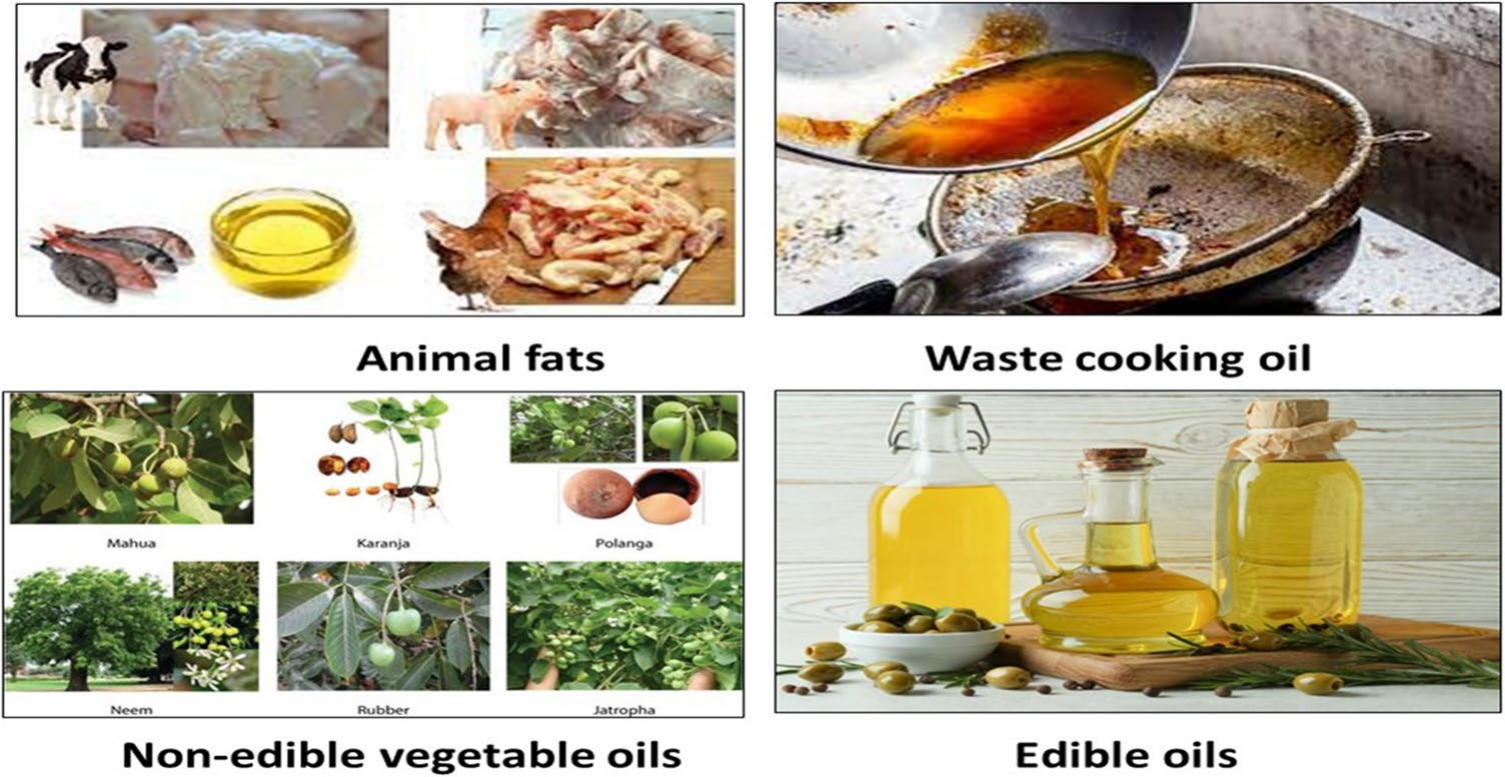
Feedstocks for biodiesel production

Biodiesel can be synthesized from various raw materials, including vegetable oils, animal fats, algae, and waste cooking oil. Each feedstock has its own advantages and challenges regarding availability, cost, sustainability, and energy content. The purpose of using different raw materials is to diversify the sources of renewable energy and reduce dependence on fossil fuels. At least 80% of the current costs of producing biodiesel come from the feedstock (Sayed and El-Gharbawy 2016). Given the global food crisis, almost 95% of biodiesel produced worldwide is manufactured from edible oils, which are viewed as unnecessary (Azizian and Kramer 2005). As a result, making biodiesel from inexpensive, non-edible oil is the current trend (Balat 2011). A few feedstocks, including palm, jatropha, microalgae, coconut tallow, and used cooking oil, stand out for their high productivity in the manufacturing of biodiesel (Gui et al. 2008). Figure 1 represents some different feedstocks for biodiesel production.

The choice of raw materials has a significant impact on the production of biodiesel in terms of yield, quality, cost, and environmental sustainability. The fatty acid profile of the raw material plays a crucial role in determining the properties and performance of biodiesel. Oils with high levels of saturated fatty acids produce biodiesel with better cold flow properties, while oils with high levels of unsaturated fatty acids produce biodiesel with better oxidative stability (Suzihaque et al. 2022). FFA content significantly impacts the reaction rate and biodiesel yield. Higher FFA content requires pre-treatment steps, such as esterification, to reduce acidity and prevent soap formation, which can hinder the reaction and reduce biodiesel purity (Javidialesaadi and Raeissi 2013). The amount of water in the raw material can also affect the transesterification reaction. Excess water can hydrolyze the fatty acid methyl esters produced during the reaction, reducing biodiesel yield. Therefore, feedstocks with low water content are preferred (Atadashi et al. 2012). The overall quality of the raw material, including its purity and the presence of impurities, can influence biodiesel production. Impurities like phospholipids, gums, and waxes can interfere with the reaction and complicate downstream processing (Manzanera et al. 2008). The environmental impact of raw material sourcing should be considered. Using waste cooking oil or non-edible oils reduces the competition for land with food production and minimizes the environmental footprint of biodiesel production. The price of the raw material is a major factor affecting the economics of biodiesel production. Utilizing inexpensive feedstocks, such as waste cooking oil or locally produced oils, can significantly reduce production costs. The choice of raw material may necessitate adjustments in processing conditions, such as reaction temperature, catalyst concentration, and reaction time, to optimize biodiesel yield and quality. Byproducts generated during biodiesel production, such as glycerol, can be utilized for various applications, adding value to the overall process and reducing waste. The sourcing of raw materials can have social and economic impacts on local communities. Promoting the use of locally available or waste-based feedstocks can stimulate agricultural economies and create employment opportunities (Srivastava et al. 2018; Suzihaque et al. 2022). In conclusion, the selection of raw materials for biodiesel production requires a careful assessment of their fatty acid profile, FFA content, water content, overall quality, environmental sustainability, cost-effectiveness, and potential for byproduct utilization. By considering these factors, biodiesel production can be optimized for yield, quality, cost, and environmental sustainability. Devaraj et al. (2020) created biodiesel from used frying oil. At 75 °C, 1 wt% catalyst concentration, 1:6 oil-to-methanol molar ratio, 350 rpm, and 90 min, 97% of the possible biodiesel output was produced. In the pilot plant, biodiesel was also produced under these process conditions, with a 97% yield. Hamed et al. (2021) converted Afia waste cooking oil (AWCO) into biodiesel fuel. At the ideal working conditions of 60 °C reaction temperature, 3 h reaction time, and 0.4 catalyst concentration, the maximum conversion and yield of biodiesel are 97.54 and 94.935%, respectively. Roy et al. (2021) reported on the conversion of used frying oil into biodiesel to manage liquid waste. The optimum conditions for the transesterification of WFO were 1:16 oil to methanol by weight, 3 wt% catalyst, 65 °C reaction temperature, and 35 min reaction time. FAME conversion (99.5%) and 96% yield are achieved at this optimal reaction setting. Karmakar and Halder (2021) researched the transesterification reaction used in supercritical settings to produce biodiesel fuel from fish waste oil. According to calculations, the experimental yield of biodiesel generation under ideal conditions was 94.6%. Al Hatrooshi et al. (2020) created the fatty acid methyl ester from waste shark liver oil (WSLO). At a methanol-to-WSLO ratio of 10.3 M, a reaction duration of 6.5 h, a temperature of 60 °C, and a catalyst concentration of 5.9 wt%, acid-catalyzed WSLO transesterification achieved 99% FAME conversion. A biodiesel yield of 99.73% was achieved at 60 °C for 1.5 h using a 1.2% catalyst and a 6:1 methanol:oil ratio. In a batch-stirred reactor, Nisa et al. (2020) conducted an experiment to produce biodiesel from the microalgae *Spirulina* sp. using 1 wt% (w/w) of palm oil as a co-solvent for methanol and potassium hydroxide. At 60 °C, with a methanol-to-palm oil molar ratio of 10:1 and a palm oil-to-microalgae weight ratio of 5:1, the best biodiesel production of 85.28% was generated. Olubunmi et al. (2020) converted the bio-oil from dairy scum waste into biodiesel. Using a 9:1 methanol-to-oil molar ratio, 40 min for the reaction, 65 °C for the reaction temperature, and 300 rpm for the mixing speed, the maximum biodiesel yield of 94.8% was achieved. In India, Jain and Sharma (2010) utilized Jatropha oil for the manufacture of biodiesel under the ideal conditions of a 3:7 (v/v) methanol-to-oil ratio and a 1% (w/w) catalyst concentration for mixing at 400 rpm with H_2_SO_4_ and NaOH. The transesterification of pretreated JCO yielded the highest yield of 90.1%. Abdulrahman (2017) made a biodiesel fuel from used cooking oil and chicken fat. The highest conversion to ester was attained at a methanol-to-oil ratio of roughly 7:1 at 60 °C and an 83% yield, also Al-Mawaali et al. (2023) utilized discarded animal fats and used cooking oil as a cheap source of feedstock to produce biodiesel. With NaOH acting as a catalyst, the discarded frying oil produced the highest yield of synthesized biodiesel (80.6%), followed by a mixture of waste cooking oil and animal fats (79.3%). Finally, using multiple raw materials as feedstock for biodiesel synthesis helps to diversify energy sources, reduce dependency on fossil fuels, and promote sustainability. Every feedstock source has benefits and drawbacks, and the choice of feedstock is influenced by factors like cost, sustainability, availability, and the needs of the biodiesel production process. Continued R&D efforts are aimed at enhancing the efficiency, cost-effectiveness, and sustainability of biodiesel production from diverse feedstocks.

### Feedstock preparation

- Cleaning and drying: The feedstock is cleaned to get rid of pollutants, water, and debris. It could entail processes like sedimentation or filtration. The feedstock is dried to eliminate any last traces of moisture after cleaning (Sait et al. 2022).
- Pretreatment: Pretreatment of feedstocks may be necessary in some situations to enhance their quality and appropriateness for the manufacture of biodiesel. Degumming, acid esterification, or neutralization are some examples of pretreatment techniques used to get rid of pollutants, gums, and free fatty acids (Singh et al. 2011).

The most popular method of treatment for reducing FFAs is acid esterification. One efficient method for lowering FFAs is glycerolysis, also known as glycerol esterification (Elgharbawy et al. 2021). Hayyan et al. (2011) treated the high FFA percentage in palm oil using sulfuric acid. They used H_2_SO_4_ by 0.75 wt%, a methanol-to-oil molar ratio of 8:1, 60 min of reaction time, and 60 °C to successfully reduce the FFA content from 23 wt% to less than 2 wt%. Kara et al. (2018) examined how various methanol-to-oil molar ratios affected the final FFA% throughout the esterification reaction while maintaining constant values for other parameters. Within 160 min of the reaction, the ideal conditions were reached at a molar ratio of 15:1. The highest conversion of 92.6% was achieved, while the FFA content decreased from 21 to 1.5%. Sadaf et al. (2018) investigated how three acids HCl, H_2_SO_4_, and H_3_PO_4_ affected used cooking oil containing 2.75 wt% FFAs. At 60 °C and a 2.5:1 methanol-to-oil molar ratio, the FFA dropped to 0.33 wt%, indicating that H_2_SO_4_ was the most effective catalyst. Sousa et al. (2010) treated castor oil with a non-catalytic glycerolysis procedure with a glycerol-to-oil mass ratio of 1:1 and an FFA of 2.5 wt% for 2 h at 120 °C. They were able to reduce the FFAs from 2.5 to 0.2 wt%.

### Triglyceride content in different feedstocks

Triglycerides are the primary component of biodiesel and are present in various amounts in the feedstocks used in the process. Mono- and diglycerides, free fatty acids, phosphatides, sterols, fatty alcohols, fat-soluble vitamins, and other compounds are among the minor constituents. The amount of triglycerides in the feedstock has a significant impact on the output and caliber of biodiesel produced. Most dietary fats and oils are mostly composed of triglycerides.

#### Vegetable oils

Various vegetable oils, including canola, sunflower, soybean, and palm oils, are frequently utilized as feedstocks in the manufacturing of biodiesel. High concentrations of triglycerides, which are made up of three fatty acid chains joined to a glycerol backbone, are found naturally in these oils. Vegetable oils, therefore, have a high triglyceride concentration, usually more than 90% by weight. Vegetable oils are ideal feedstocks for the synthesis of biodiesel because of their high triglyceride content. Because refined vegetable oils convert pure triglycerides (TG) to FAME at a high rate and quickly, they are the ideal feedstock for producing biodiesel.

#### Animal fats

Fish oil, tallow, lard, and chicken fat are examples of animal fats that can be utilized as feedstocks to produce biodiesel. Comparing animal fats to vegetable oils, the former usually have a lower triglyceride concentration, usually between 70 and 85% by weight. The species of the animal, its nutrition, and the rendering techniques all affect the variation in triglyceride content. Animal fats can still be effectively turned into biodiesel even when their triglyceride level is slightly lower.

#### Waste oils and greases

The manufacturing of biodiesel can use recycled waste cooking oils and greases from food processing industries, restaurants, and other sources as feedstocks. Depending on their quality and place of origin, waste oils and greases might include varying amounts of triglyceride. The quality and conversion efficiency of biodiesel can be impacted by the presence of contaminants or elevated quantities of free fatty acids in these feedstocks.

#### Algae and microorganisms

The capacity of algae and specific microorganisms to accumulate large concentrations of lipids (fatty acids and triglycerides) makes them viable feedstocks for the generation of biodiesel. Depending on the species and growing environment, triglycerides can make up a large portion of algal oils, anywhere from 20 to 60% by weight. Table 1 represents the triglyceride content of different feedstocks.

**Table 1:** Triglyceride content in different feedstocks

Feedstock	Triglyceride content	References
Soybean oil	96.8	Li et al. (2018)
Palm oil	99.4	Ali et al. (2019)
Sunflower oil	99.3	Liu et al. (2011)
Cottonseed oil	98.1	Liu et al. (2011)
Waste cooking oil	97.5	Li et al. (2018)
Chicken fat	91.4	Alptekin et al. (2014)
Waste fish fat	87.2	Mrad et al. (2012)
Microalgal oil	99	Çakırca et al. (2019)

## Biodiesel as a promising alternative source of biofuel

Among the emerging alternatives, biodiesel has garnered significant attention as a promising biofuel with the potential to reduce reliance on petroleum-based fuels and mitigate environmental impacts. The non-toxic, biodegradable fuel known as biodiesel is made from leftover cooking oil, animal fats, or vegetable oil, and presents several advantages over conventional diesel. Firstly, its renewable nature alleviates concerns over depleting fossil fuel resources. Unlike petroleum diesel, which is extracted from finite underground reservoirs, biodiesel can be produced from continuously replenished sources, ensuring long-term sustainability. Secondly, biodiesel boasts superior environmental credentials compared to diesel. Its burning emits significantly lower degrees of particulate matter, nitrogen oxides, sulfur oxides, and carbon monoxide, all of which contribute to air pollution and its detrimental effects on human health and the environment. Additionally, biodiesel’s lower greenhouse gas emissions make it a promising alternative for reducing the transportation sector’s contribution to climate change (Demirbas 2009). Moreover, biodiesel offers economic benefits, particularly for regions with abundant feedstock sources. Local production of biodiesel can stimulate agricultural economies, reduce reliance on imported petroleum, and create employment opportunities. Additionally, biodiesel’s compatibility with existing diesel engines eliminates the need for expensive infrastructure overhauls, facilitating a smoother transition toward a more sustainable fuel source (Fukuda et al. 2001). Despite its promise, biodiesel faces certain challenges that need to be addressed for its widespread adoption. One challenge lies in its economic viability. The production cost of biodiesel is currently higher than that of petroleum diesel, primarily due to feedstock costs and processing expenses. However, advancements in production technologies and economies of scale are expected to reduce biodiesel costs over time.

Another challenge involves the availability of suitable feedstocks. While various plant oils, such as soybean, palm, and jatropha, can be used for biodiesel production, concerns have arisen regarding land-use competition for the food industry and the potential environmental effects of broad-scope palm oil plantations. Sustainable feedstock sourcing strategies, such as utilizing waste cooking oil and cultivating oil crops on marginal lands, are crucial to addressing these concerns (Knothe 2010; Singh and Singh 2010). In conclusion, biodiesel presents a compelling alternative to conventional diesel, offering a combination of sustainability, environmental benefits, and economic potential. Addressing the current challenges related to feedstock availability and production costs will pave the way for biodiesel’s wider adoption and its significant contribution to a more sustainable energy future.

### Innovations in biodiesel applications

**Fig. 2:**
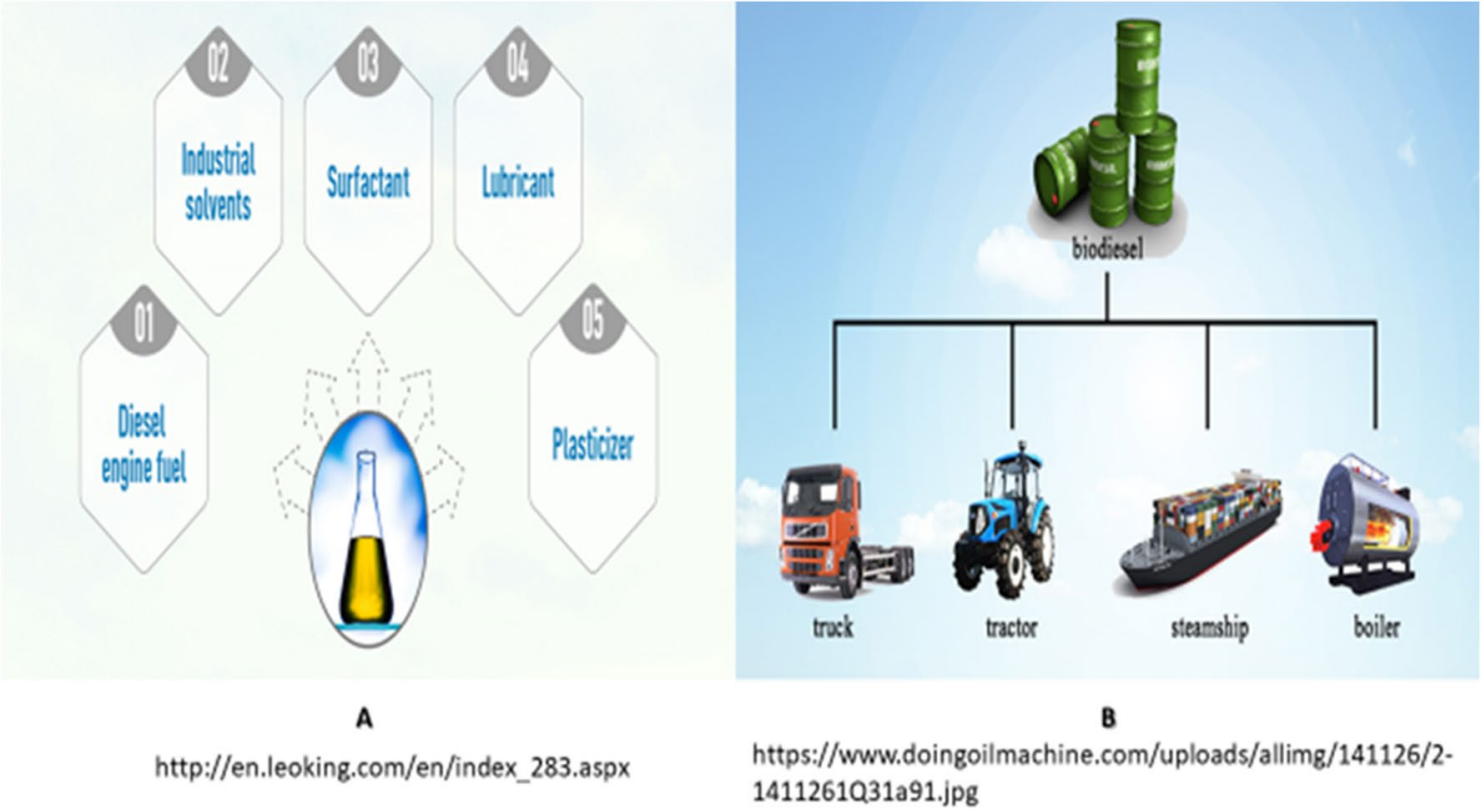
Biodiesel applications

Biodiesel, a renewable and cleaner-burning alternative to conventional diesel fuel, has gained significant attention and widespread applications in recent years. Its versatile nature allows for various uses across different sectors. In transportation, biodiesel can be blended with petroleum diesel to power vehicles, reducing emissions of greenhouse gases and air pollutants. Moreover, biodiesel finds applications in industrial settings, where it serves as a substitute for petroleum-based fuels in machinery and equipment. The growing interest in sustainable energy solutions has spurred research and development efforts, leading to advancements in biodiesel production techniques, feedstock selection, and engine compatibility. Recent studies (Mobin et al. 2022; Tripathi et al. 2023) have explored the potential of advanced feedstocks, such as algae and waste oils, to enhance biodiesel production efficiency and reduce environmental impacts. These advancements in biodiesel applications and technology are crucial steps in achieving a more sustainable and greener energy future. Figure 2 shows some of the applications of biodiesel.

## Transesterification in biodiesel production

Alcohol and lipids react chemically to produce fatty acid alkyl esters, a process known as transesterification. Triglycerides and alcohol are transesterified to produce FAAE and glycerol. Triglycerides and alcohols combine to form diglycerides in the first stage, which are then converted to monoglycerides and glycerol, each of which yields an alkyl ester (Thangaraj et al. 2019). Among the factors influencing biodiesel yield in transesterification are time, temperature, type and concentration of the catalyst, kind of feedstock oil, and alcohol-to-oil ratio. It is possible to reverse the transesterification process. To shift the reaction’s equilibrium in favor of the product’s production, an excess of alcohol is therefore necessary. Alcohols with short chains, long chains, and cyclic chains are all used in this process. The availability, polarity, better reactivity, and inexpensive price of methanol and ethanol, however, make them popular choices (Avhad and Marchetti 2015), Fig. 3 illustrates the characteristics and features of the transesterification process used to make biodiesel, and Fig. 4 shows a schematic representation of the biodiesel synthesis path through the transesterification process.

**Fig. 3:**
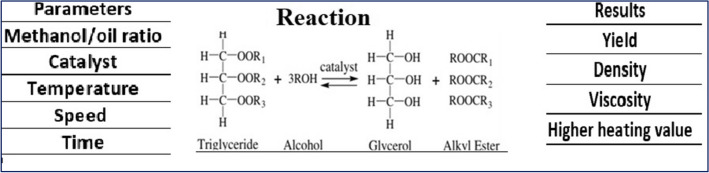
The characteristics and features of the transesterification process used to make biodiesel

**Fig. 4:**
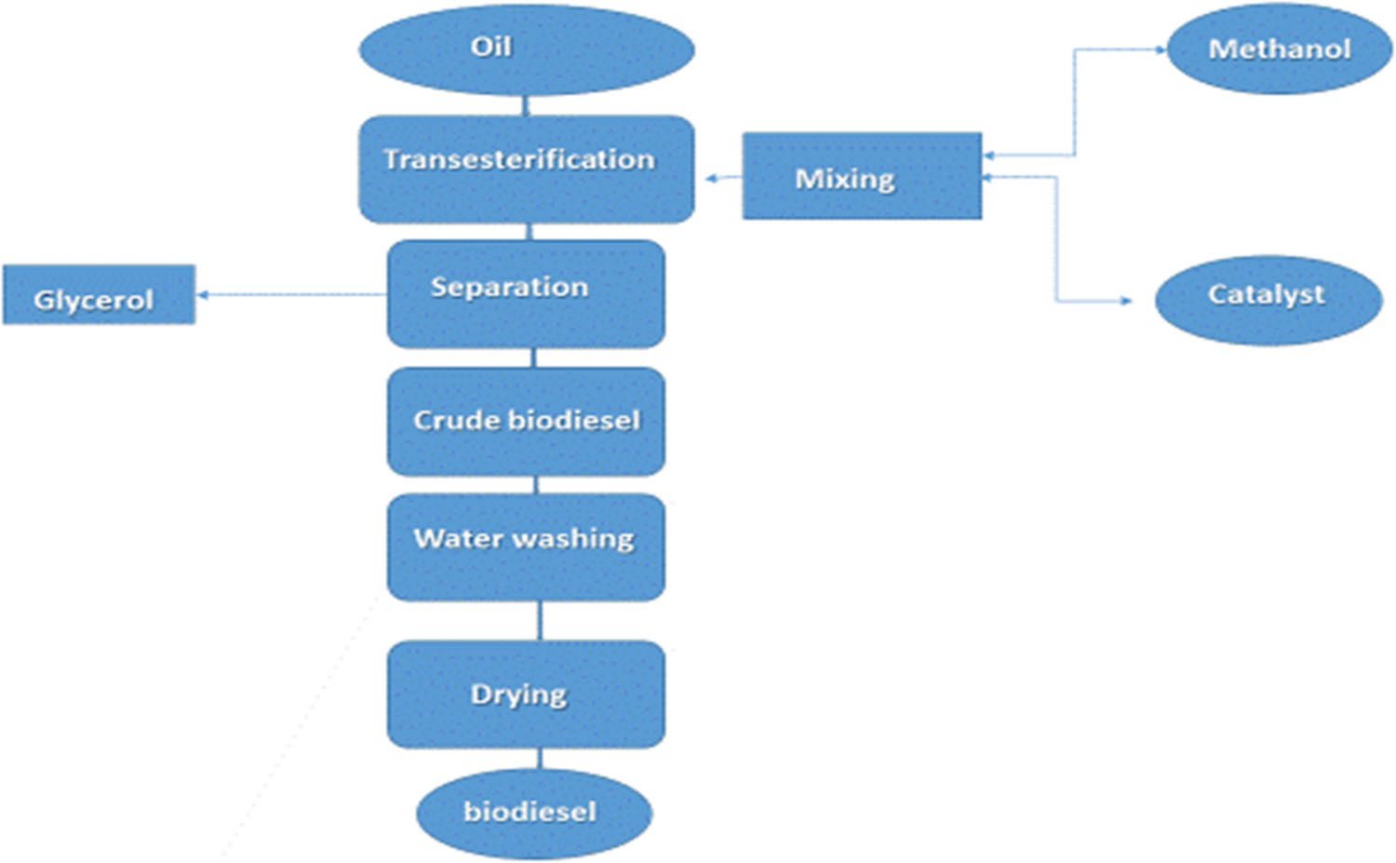
Schematic representation of the biodiesel synthesis path through the transesterification process

**Fig. 5:**
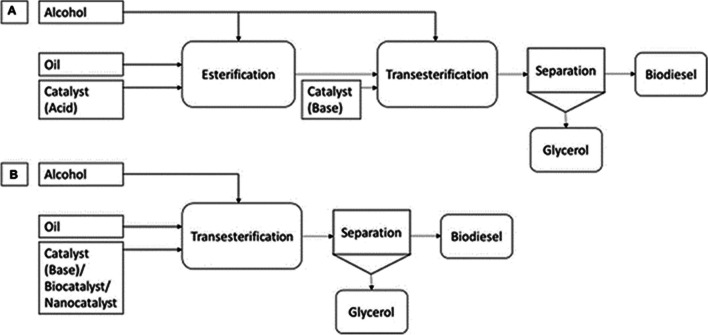
Biodiesel manufacturing can be done in two steps (**A**) or one step (**B**) (Fattah et al. 2014; Mofijur et al. 2014)

Triglycerides are changed into diglycerides, monoglycerides, and finally glycerol by a sequence of chemical events called transesterification, which is the process used to produce biodiesel. Alkali catalysts are usually used in a single phase of this process. However, a two-step procedure can be required if the feedstock has large concentrations of water or free fatty acids (FFAs). Fatty acid esters, or acid-catalyzed alcoholysis, are the initial step that turns FFAs into fatty acid esters. The process of transesterification, which turns the fatty acid esters into biodiesel, comes next. Figure 5 displays the schematic diagram for the manufacture of biodiesel in both one and two steps.

**Fig. 6:**
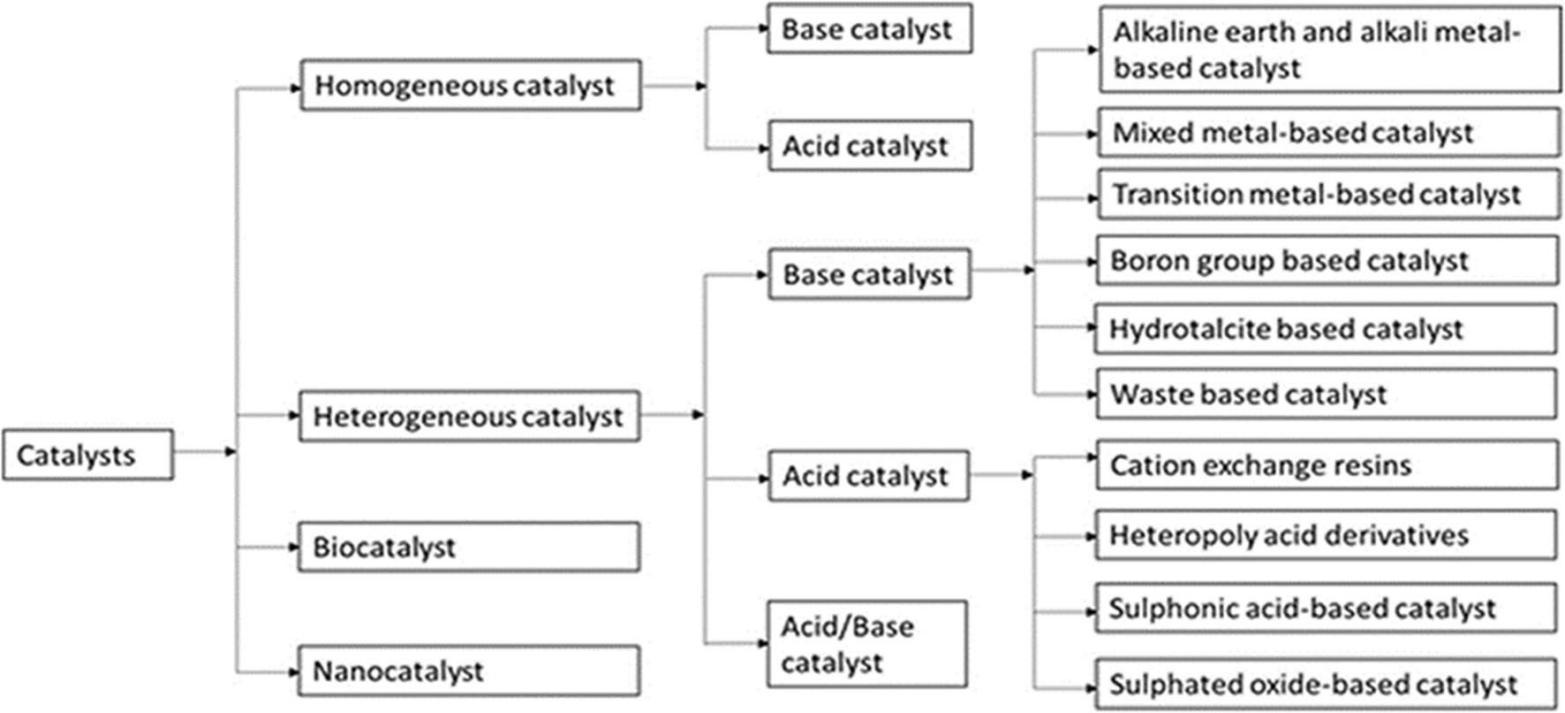
Different catalysts used in biodiesel production (Rizwanul Fattah et al. 2020)

Chemical or biological catalysts can be used in transesterification reactions that are catalyzed. The classification is shown in Fig. 6. There are both homogeneous and heterogeneous chemical catalysts. Base or acid catalysts are included in the homogeneous catalyst. The heterogeneous catalyst is made up of nano-, biomass waste-based, base, and acid–base functionalities (Thangaraj et al. 2019). The choice of any catalyst is influenced by the following factors: oil quality, FFA content, operating conditions, necessary catalyst activity, cost, and availability (Tacias-Pascacio et al. 2019). The advantages and disadvantages of different catalysts are presented in Table 2.

**Table 2:** Pros and cons of utilizing various catalysts in the process of transesterification

Catalysts	Pros	Cons	References
Homogeneous:	• They can facilitate faster reaction rates and higher conversion yields compared to other catalyst types (such as heterogeneous catalysts) due to their ability to uniformly distribute throughout the reaction mixture. This homogeneous distribution enables better contact between the catalyst and reactants, resulting in improved reaction kinetics and the overall efficiency of the process • Exhibit greater selectivity, promoting the desired transesterification reaction while minimizing unwanted side reactions. This selectivity contributes to the higher purity and quality of the biodiesel product • Versatility and adaptability to various feedstocks. They can effectively catalyze the transesterification of different types of triglycerides, expanding the range of potential biodiesel sources • Operate under milder reaction conditions, such as lower temperatures and pressures, which can result in energy savings and reduced production costs	• One significant drawback is their potential for leaching into the biodiesel product, which can complicate the separation and purification processes. Homogeneous catalysts, being soluble in the reaction mixture, can remain in the biodiesel phase even after the completion of the transesterification reaction. This can increase the difficulty and cost associated with removing the catalyst from the final product, leading to additional purification steps • Some homogeneous catalysts used in biodiesel production may have limited stability and reusability. Catalyst deactivation or degradation can occur over time, reducing their effectiveness and necessitating frequent replacement or regeneration. The need for catalyst regeneration or disposal can add to the overall cost and environmental impact of the biodiesel production process • Impurities, including water, free fatty acids, and glycerol, that are present in the feedstock may cause certain homogeneous catalysts to become sensitive. These impurities can inhibit the catalytic activity or lead to unwanted side reactions, necessitating extra pre-treatment steps to ensure high-quality biodiesel production	Meher et al. (2006); Demirbas (2008); Kumar (2021)
Heterogeneous:	• Easy separation: Heterogeneous catalysts, being solid materials, can be separated from the reaction mixture with ease. This characteristic simplifies the purification process and reduces the costs associated with catalyst recovery. The solid catalysts can be easily filtered or decanted, permitting effective catalyst and biodiesel product separation • Catalyst reusability: Heterogeneous catalysts generally exhibit good stability and can be repeatedly utilized without experiencing a noticeable decrease in activity. This reusability feature reduces the overall catalyst cost and enhances the profitability of the manufacture of biodiesel • Tolerance to impurities: Heterogeneous catalysts often demonstrate higher tolerance to impurities commonly found in feedstocks. This tolerance eases the overall process of producing biodiesel, potentially lowering production costs, and eliminating the need for extensive pre-treatment of the feedstock • Environmental sustainability: Heterogeneous catalysts are considered environmentally friendly due to their reduced potential for leaching into the biodiesel product and lower toxicity compared to some homogeneous catalysts. This characteristic aligns with the goal of sustainable biodiesel production	• Mass transfer limitations: Heterogeneous catalysts often exhibit slower reaction rates compared to homogeneous catalysts due to mass transfer limitations. The reactants need to diffuse through the liquid phase to reach the catalyst surface, which can result in reduced reaction kinetics and longer reaction times • Catalyst deactivation: Heterogeneous catalysts can experience deactivation or loss of activity over time, primarily due to the formation of surface contaminants or catalyst poisoning. Impurities present in the feedstock, such as water, free fatty acids, and glycerol, can interact with the catalyst and inhibit its activity, leading to decreased performance and the need for catalyst replacement or regeneration • Complex catalyst preparation: The preparation of heterogeneous catalysts can be more complex compared to homogeneous catalysts. It may involve multiple steps, such as synthesis, activation, and modification, which can increase the production cost and require specialized expertise • Limited catalyst selectivity: Some heterogeneous catalysts may exhibit lower selectivity, leading to the production of unwanted byproducts or side reactions, which may have an impact on the biodiesel product's quality and purity	Freedman et al. (1986); Meher et al. 2006; Balat (2011)
Enzymatic:	• Mild reaction conditions: Enzymes typically operate under mild reaction conditions, including lower temperatures and atmospheric pressure. This mildness reduces energy requirements and can lead to cost savings in the biodiesel production process • High specificity and selectivity: Enzymes exhibit high specificity towards the targeted reactions, promoting the desired transesterification while minimizing side reactions. This selectivity contributes to the higher purity and quality of the biodiesel product • Tolerance to impurities: Enzymatic catalysts often demonstrate higher tolerance to impurities present in the feedstock, such as water and free fatty acids, compared to other catalyst types. This tolerance reduces the need for extensive pre-treatment of the feedstock, simplifying the overall biodiesel production process and potentially lowering production costs • Biodegradability and environmental sustainability: Enzymes are biodegradable and environmentally friendly catalysts. They can be derived from renewable sources and offer the advantage of reduced environmental impact compared to traditional catalysts • Catalyst reusability: Enzymes can be immobilized on solid supports, allowing for their repeated use in multiple batches of biodiesel production. This reusability feature reduces the overall catalyst cost and contributes to the economic viability of the process	• Higher cost: Enzymes, especially those derived from microbial sources, can be expensive compared to other catalyst types. The costs of enzyme production, purification, and immobilization can significantly contribute to the overall cost of biodiesel production • Sensitivity to Reaction Conditions: Enzymes are sensitive to reaction conditions such as temperature, pH, and water content. The optimal conditions for enzyme activity may differ from the ideal conditions for transesterification, requiring careful control and optimization of the reaction parameters. Any deviation from the optimal conditions can result in reduced enzyme activity and lower biodiesel yields • Limited operational stability: Enzymes can experience operational stability issues during prolonged use. Factors such as enzyme denaturation, microbial contamination, and enzyme leaching from the immobilization matrix can lead to decreased enzyme activity over time, necessitating enzyme replacement or regeneration • Longer reaction times: Enzymatic transesterification reactions often require longer reaction times compared to other catalyst types. This is primarily due to the slower reaction kinetics associated with enzymatic catalysis, which can impact the overall productivity and efficiency of the biodiesel production process	Du et al. (2004); Xie and Wang (2014)

### Homogeneous catalysis

Homogeneous catalysts are the ones that are most frequently used in the production of biodiesel because they are simple to use and require less time to complete a reaction. When dissolving a homogeneous catalyst, a solvent that is in the same phase as all the reactants is usually utilized (Rizwanul Fattah et al. 2020). Bhuana et al. (2020) used leftover beef tallow and methanol as solvents, in a KOH catalyst to create biodiesel. Ethanol effectively functioned as a co-solvent, lowering reaction time by 61.11% and functioning as a low-polarity active ester exchange agent, which prevented soap formation and increased yield by 3.08%. Karmee et al. (2015) looked at the transesterification of algal oil using methanol as the solvent. Under identical reactional conditions, HCl outperformed the homogeneous H_2_SO_4_ catalyst. Rice bran oil was esterified by Arora et al. (2015) utilizing sulfuric acid as a uniform catalyst. Studies have been done on how the oil-to-methanol molar ratio (1:5 to 1:30), catalyst concentration (0.15 to 1.0 wt%), and reaction temperature (45 to 60 °C) affect the conversion of FFA. Siddiqua (2015) transesterified palm oil to create biodiesel. Further observation shows that a mixture of methanol and NaOH at a reaction temperature of 55 °C produced the highest production. Abdulsalam (2023) assessed the process of turning thevetia peruviana seed oil into biodiesel using two different catalysts (NaOH and KOH). Separate NaOH and KOH pellets were dissolved in methanol to create different catalysts with different amounts of sodium and potassium methoxide, respectively. The NaOH catalyst’s conversion yield was 81.2%, 80.4%, and 89%, whereas the KOH catalyst’s conversion yield was 96.8%, 88.4%, and 84.0. Through the calcination of leftover pineapple leaves, de Barros et al. (2020) created a unique catalyst. After a 30-min reaction period at 60 °C, 4 wt% of catalyst, a molar ratio of 1:40 for oil to methanol, and an oil-to-biodiesel conversion of above 98%, a high catalytic activity was detected. This activity is likely connected to the 85-wt% presence of alkali/alkali metals (K, Ca, and Mg). Kasirajan (2021) used two-step procedures to produce biodiesel from *Chrysophyllum albidum* seed, a non-edible source. Transesterification is performed on oil from *Chrysophyllum albidum* seeds after esterification employing a homogeneous catalyst of H_2_SO_4_. The maximum oil-to-biodiesel conversion was 99.2 wt% when the ideal situations were achieved, which included a 1:9 oil-to-methanol molar ratio, 1 wt% KOH, 500 rpm, and 40 min at 65 °C. Belkhanchi et al. (2021) showed that transesterifying used frying oils (UFO) at 18 °C for 60 min of reaction in the presence of methanol using MeOH/UFO 6:1 mol proportion and 1 wt% of KOH yields the best conversion of UFO. Jain et al. (2023) gave a description of the homogeneous base catalyst-based single-step transesterification process for producing biodiesel from waste cooking oil that contains high levels of free fatty acids and algal oil. According to the findings, a biodiesel yield of 92% may be achieved under optimal conditions, which include a 1.5% catalyst (w/w), a methanol to oil ratio of 21:1, a time of 110 min, and a temperature of 50 °C. Saeed et al. (2021) investigated *S. elongata* algal for biodiesel creation. To evaluate transesterification to FAME, zeolitic catalysts, KOH, and HCl were used. KOH produced the maximum biodiesel yield (99.9%), which was obtained under the ideal reaction conditions of a 1.0% catalyst, 60 °C, 4 h, and a 1:4 volume ratio between oil and methanol. Table 3 highlights some of the recently published research on the use of several homogeneous catalyst types for biodiesel synthesis, various feedstock sources, experimental setups, and biodiesel yields.

**Table 3:** Different types of homogeneous catalysts used for biodiesel synthesis

Type of feedstock	Homogeneous catalyst	Experimental conditions Temperature (°C) M:O molar ratio Catalyst (wt. %) time (h)	Biodiesel yield (%)	References
***Elaeagnus angustifolia L*** oil	Potassium hydroxide	60 °C -9:1–1%-1 h	95	Kamran et al. (2020)
Waste shark liver oil	H_2_SO_4_	60 °C-10:1–5.9%-6.5 h	99	Al Hatrooshi et al. 2020
Cotton oil	KF/bentonite	120 °C-13:1–6%-6 h	95	da Costa and de Andrade Lima (2021)
***Bauhinia variegata*** seeds oil	KOH	60°C-10:1–0.6%-0.5 h	99	Perumal and Mahendradas (2022)
Waste frying vegetable oil	KOH	60 °C-12:1–1.5%-1.5 h	97	OA et al. (2019)
Tall oil fatty acids	H_2_SO_4_	55 °C-15:1–0.5%-1 h	96.76	Lawer-Yolar et al. (2021)
Waste cooking oil	NaOH	40 °C-9:1–1%-2 h	98.22	Abdel-Hamid et al. (2023)

### Heterogeneous catalysis

Heterogeneous catalysts go through different phases or states than reactants. According to Melero et al. (2009), these are the catalysts that often generate active sites when reacting with their reactants. Greater oil/alcohol ratios and greater temperatures than in homogeneous catalysis are the primary disadvantages of this catalysis. The catalyst’s improved reusability and ease of separation and purification are other advantages. Mohamed et al. (2020) prepared by quickly pyrolyzing rice straw, a heterogeneous catalyst (RS-SO_3_H) was created. The yield of biodiesel was 90.37%. in ideal conditions: 20:1 methanol: oil molar ratio with a 10% catalyst at 70 °C for 6 h. Choksi et al. (2021) created a solid acid catalyst using the sulfonation carbonization process from a palm fruit bunch. After that, the catalyst was put through esterification and transesterification processes to produce biodiesel. Utilizing a 4% catalyst, a 21:1 methanol-to-oil molar ratio, and a 60 °C temperature, an optimal yield of 88.5 wt% methyl ester was obtained in 180 min. Aghel et al. (2019) wanted to improve a pilot-scale microreactor that used kettle limescale to turn used cooking oil (WCO) into biodiesel. The produced biodiesel had a maximum conversion of 93.41% at 61.7 °C, a catalyst concentration of 8.87 wt %, a methanol-to-oil 1.7:3 volumetric ratio, and 15 min. Bhatia et al. (2020) developed a heterogeneous catalyst to initiate the transesterification of used cooking oil by pyrolyzing waste cork. The greatest conversion (98%) for the heterogeneous catalyst produced at 600 °C occurred at alcohol:oil ratios of 25:1, catalyst loadings of 1.5% w/v, and temperatures of 65 °C. Sahani et al. (2019) used a solid-base catalyst called barium cerate in the transesterification procedure to produce biodiesel from Karanja oil. To synthesize perovskite barium cerate with maximum phase purity, the calcination temperature was optimized. At 1.2 wt% catalyst, 1:19 oil-to-methanol molar ratio, 65 °C, 100 min, and 600 rpm, karanja oil methyl ester with 98.3% conversion was obtained. Kamel et al. (2019) utilized the fig leaves that had undergone calcination, KOH activation, and activation. The highest conversion to biodiesel (92.73%) was obtained from fig leaves treated with KOH under ideal conditions (2 h of heating, a 6:1 alcohol/oil molar ratio, 1% catalyst, and 400 rpm). Singh et al. (2023) produced biodiesel from *Jatropha curcas* oil using the transesterification technique and calcium oxide. The results of the experiment demonstrate that at a methanol/oil ratio of 12:1, 65 °C, 3 h, and a catalyst concentration of 5 wt%, a biodiesel yield of 81.6% was produced. Carbon spheres were the heterogeneous acid catalyst that Nata et al. (2017) utilized. A maximum yield of 87% was achieved at 60 °C and 1 h when WCO was used as the feedstock to make biodiesel utilizing a C–SO_3_H acid catalyst. Du et al. (2019) converted *Scenedesmus quadricauda* algal oil into biodiesel using a cobalt-doped CaO catalyst. Cao was obtained from eggshells and calcined at 400, 700, and 900 °C. Todorović et al. (2019) conducted research on canola oil-based potassium-supported TiO_2_ for biodiesel generation. At 55 °C for 5 h, with a 6 wt% catalyst and a 54/1 methanol/oil, the highest biodiesel output of > 90% was discovered. Salinas et al. (2012) created a carbon-based MgO catalyst for castor oil transesterification utilizing the sol–gel method. With a 96.5% biodiesel output at 6 wt% catalyst loading and a 12:1 ethanol/oil ratio at 75 °C for 1 h, the MgO/UREA-800 demonstrated remarkable catalytic activity. Gardy et al. (2019) made a strong, magnetic core–shell SO_4_/Mg–Al–FeO_3_ heterogeneous catalyst with the use of surface functionalization, encapsulation, and stepwise coprecipitation. Utilizing the synthesized catalyst, the transesterification reaction was carried out with the highest possible yield of 98.5% at 9:1 methanol/WCO, 95 °C, and 5 h. Table 4 highlights some of the recently published research on the use of several heterogeneous catalyst types for biodiesel synthesis, various feedstock sources, experimental setups, and biodiesel yields.

**Table 4:** Different types of heterogeneous catalysts used for biodiesel synthesis

Type of feedstock	Heterogeneous catalyst	Experimental conditions Temperature (°C) M:O molar ratio Catalyst (wt. %) time (h)	Biodiesel Yield (%)	References
Soybean oil	Potassium methoxide	80 °C-6:1–2%-0.25 h	91	Celante et al. (2018)
***Parkia biglobosa*** oil	Clay-Na_2_CO_3_	60 °C-12:1–2%-1.5 h	94.7	Takase et al. (2023)
***Ricinus communis***	Na_2_ZrO_3_	65 °C-15:1–5%-3 h	99.9	Martínez et al. (2018)
Mixture of crop mustard and edible waste oil	Calcium oxide catalyst prepared from fish bones	55 °C-12:1–0.3%-5 h	94.95	Abbas Ghazali and Marahel (2022)
Soybean oil	*M. acuminata* banana trunk ash (MBTA)	25 °C-6:1–0.07%-6 h	98.39	Rajkumari and Rokhum (2020)
Waste cooking oil	12-molybdophosphoric acid	190 °C-90:1–5%-4 h	94.5	Gonçalves et al. (2021)
Palm oil	Zinc oxide supported silver nanoparticles	60 °C-10:1–10%-1 h	97	Laskar et al. (2020)
Palm fatty acid distillate	Tea waste	65 °C-9:1–4%-1.5 h	97	Rashid et al. (2019)

### Enzyme-based catalyst

Enzyme-based catalysts are produced from living things that speed up reactions while maintaining the stability of their composition (Amini et al. 2017). Extracellular lipases are the enzymes that have been isolated and processed from the microbial broth. In contrast, intracellular lipase remains inside the cell or in its walls of production (Gog et al. 2012). One drawback of employing extracellular enzymes as catalysts is the expense and difficulty of the separation and purification procedures (Rizwanul Fattah et al. 2020). The efficiency of the bio-catalyzed transesterification process is influenced by the enzyme’s source and the process variables (Aransiola et al. 2014). Enzymatic biodiesel production also has the advantages of being simple to remove, operating at a temperature between 35 and 45 °C, producing no byproducts, and allowing catalysts to be reused (Christopher et al. 2014). For the transesterification of low-grade fish oil, Marín-Suárez et al. (2019) used Novozym 435 lipase; the greatest FAEE yield was 82.91 wt% after 8 h, 35 °C, an excess of ethanol, and 1% catalyst. Novozym 435 can be used for 10 continuous cycles with a maximum activity decrease of 16%. Jayaraman et al. (2020) studied used cooking oil enzymatic transesterification with the use of pancreatic lipase to make methyl ester. The best reaction conditions were discovered to be methanol as the alcohol 3:1 M ratio, 1.5% enzyme concentration (by weight of WCO), 4 h reaction duration, 60 °C, and 88% yield after numerous attempts. Fatty acid methyl ester (FAME) was produced by Choi et al. (2018) produced FAME from the oil in rice bran by just adding methanol. The 83.4% yield was reached after 12 days under ideal conditions.

### Nanocatalysts in transesterification

Nanocatalysts have garnered significant interest in the production of biodiesel. Because of their special qualities, which include a large active surface area, high reusability, better catalytic efficiency, high biodiesel conversion, and sustainability, nanocatalysts can be superior to conventional catalysts (Qiu et al. 2011). Since they are easily removed from the final products and retain their catalytic activity even after being reused several times, nanocatalysts are widely sought (Ahmed et al. 2023). There are numerous ways to create nanocatalysts. Among the techniques are microwave combustion, chemical vapor deposition, impregnation, and gas condensation (Quirino et al. 2016; Ambat et al. 2018). Some of the latest works on nanocatalysts for the transesterification reaction are listed in Table 5.

**Table 5:** Various nanocatalysts in biodiesel production

Feedstock	Catalyst	Experimental conditions	Biodiesel Yield (%)	References
		Temperature (°C) M:O molar ratio Catalyst (wt.%) time (h)		
Waste cooking oil	Nano CaO	60 °C-12:1–2.5%- 2 h 94		Erchamo et al. (2021)
Waste cooking oil	Sodium oxide impregnated on carbon nanotubes (CNTs)	65 °C-20:1–3%-3 h	97	Ibrahim et al. (2020)
Used cooking oil	Graphene oxide and bimetal zirconium/strontium oxide nanoparticles	120 °C-4:1–0.5%-1.5 h	91	Madhuranthakam et al. (2020)
Used frying oil	Nano CaO	50 °C-8:1–1%-1.5 h	96	Degfie et al. (2019)
Used frying oil	Nano Mgo	65 °C-24:1–2%-1 h	93.3	Ashok et al. (2018)
Sunflower oil	MgO/MgAl_2_O_4_nano-catalyst	110 °C-12:1–3%-3 h	95.7	Alaei et al. (2018)
Sunflower oil	Cs/Al/Fe_3_O_4_ nano-catalyst	58 °C-12:1–1%-2 h	94.8	Mostafa et al. (2013)
Chicken fat	CaO/CuFe_2_O_4_	70 °C-15:1–3%-4 h	94.52	Seffati et al. (2019)
Waste cooking oil	ZnCuO/N-doped graphene (NDG)	180 °C-15:1–10%-8 h	97.1	Kuniyil et al. (2021)
Olive oil	Magnetite nanoparticle-immobilized lipase	37 °C-12:1–1%-1 h	45	Amruth Maroju et al. (2023)
Microalgae oil	Fe_3_O_4_/ZnMg(Al)O solid	65 °C-12:1–3%-3 h	94	Chen et al. (2018)
Olive oil	MgO nanoparticles	60 °C-10:1–2%-2 h	80	Amirthavalli and Warrier (2019)
Tannery waste	Cs_2_O loaded onto a nano-magnetic core	65 °C-21:1–7%-5 h	97.1	Booramurthy et al. (2020)
Used cooking oil	Bifunctional magnetic nano-catalyst	65 °C-12:1–4%-2 h	98.2	Hazmi et al. (2021)
***Ulva lactuca***, a marine macroalgae	Clay with zinc oxide as nanocatalyst	55 °C-9:1–8%-0.83 h	97.43	Kalavathy and Baskar (2019)
***Calophyllum inophyllum*** oil	Zinc-doped calcium oxide nanocatalyst	55 °C-9:1–6%-1.33 h	89	Naveenkumar and Baskar (2019)
***Camelina sativa*** seed oil	MgO/Fe_2_O_3_-SiO_2_ core–shell magnetic nanocatalyst	70 °C-12:1–4.9%-4.1 h	99	Rahimi et al. (2021)

#### Metal-oxide nanocatalysts

The most popular nanocatalysts are those based on metal oxide, and they play a crucial role in maximizing the synthesis of biodiesel. Nanoparticles that will be employed for transesterification catalysis have been created using the oxidized forms of numerous different metals, including Mg, Zn, and Ca (Pandit et al. 2023). Jamil et al. (2021) created highly efficient barium oxide using catalysts made of molybdenum oxide. Optimal conditions include 12 methanol/oil, 120 min, 65 °C, and a 4.5wt% catalyst. The best yield was achieved under these conditions, which resulted in a 97.8% yield. Sahani et al. (2020) produced biodiesel with a transesterification reaction involving used cooking oil and a mixed metal oxide catalyst made of Sr–Ti. Methanol as the alcohol in an 11:1 M ratio, 1% catalyst, an 80-min reaction period, and a temperature of 65 °C with 98% FAME conversion were found to be the best reaction conditions. In a study conducted by Tayeb et al. (2023), the production of biodiesel using a CaO catalyst through the transesterification of WCO was investigated. The study determined the optimal reaction parameters to be a WCO/methanol molar ratio of 1:6, a 1% CaO nanocatalyst, a reaction temperature of 70 °C, and a reaction duration of 85 min, which resulted in a 97% biodiesel yield.

#### Carbon nanocatalysts

Nanocatalysts are created from carbon materials, including graphene and reduced graphene oxides (Nizami and Rehan 2018). Due to their diverse structural, mechanical, thermal, and biocompatibility qualities, carbon nanocatalysts are good catalysts and have advantageous applications in electrocatalytic devices such as fuel cells and other electro-processing systems. CNTs are often manufactured from graphite sheets that have been wound into cylinder forms. They have a large surface area, measure in nanometers, and are incredibly biocompatible (Rai et al. 2016).

#### Zeolite nanocatalysts

Large exterior surface areas and the hydrophobic nature of nanozeolites increase enzyme access to the substrate. Natural zeolite materials are far less frequently used in commercial industries than synthetic-based products. Commercially available synthetic zeolites such as ZSM-5, X, Y, and beta are used primarily in the production of biodiesel (Abukhadra et al. 2019). Using zeolites from NaY, KL, and NaZSM-5, Wu et al. (2013) produced CaO catalysts that were utilized to catalyze the transformation of methanol with soybean oil. In comparison to pure CaO, the activities of synthesized catalysts were studied. It was discovered that after being supported by zeolites, the CaO catalyst’s activity improved, with the CaO/NaY catalyst showing the greatest performance. Using the CaO/NaY catalyst, methanol-to-soybean oil 9:1 molar ratio at 65 °C with a reaction period of 3 h, and a 3% catalyst were used to produce a 95% biodiesel yield. Firouzjaee and Taghizadeh (2017) synthesized a CaO/NaY-Fe_3_O_4_ nano-magnetic catalyst that was employed for the generation of biodiesel. The ideal methanol-to-oil molar ratio is 8.78, the catalyst loading is 5.19% (30% CaO loaded on the surface nanomagnetic zeolite), and the reaction period is 4 h. The maximum methyl esters obtained are 95.37%.

## Distinct behavior of nanocatalysts during biodiesel production

Nanocatalysts have been widely used in biodiesel production due to their high catalytic activity, low cost, and environmental friendliness. The properties of nanocatalysts can vary depending on the preparation method, which can affect their catalytic performance. For example, the size, shape, and surface area of the catalyst particles can influence the reaction kinetics and yield of biodiesel. Recent studies have investigated the effects of different preparation methods on the properties of nanocatalysts for biodiesel production. The preparation method and calcination temperature are important factors that can affect the properties and catalytic performance of nanocatalysts for biodiesel production. Further research is needed to optimize the preparation methods and properties of nanocatalysts to improve the efficiency and sustainability of biodiesel production. We can offer general insights into the variations of nanocatalysts throughout the biodiesel production process, focusing on the following aspects.

Catalyst types: Different generations of nanocatalysts may involve distinct types of materials. For instance, first-generation nanocatalysts might include basic materials, while second- or third-generation may involve more advanced materials like metal oxides, zeolites, or other nanostructured materials.

Particle size: Advances in nanotechnology enable the control of particle size in nanocatalysts. The particle size can significantly impact catalytic activity. Smaller particle sizes may provide larger surface areas and enhanced catalytic efficiency.

Functionalization: The functionalization of nanocatalysts with specific groups or ligands can vary across generations. Functionalization can influence the catalyst’s selectivity and stability during biodiesel production.

Reusability and stability: Reusability and recovery are the two main advantages of using heterogeneous nanocatalysts in the production of biodiesel. The nanocatalyst is recovered and utilized again at each stage of these processes, which include many cycles of producing biodiesel. Nanocatalysts are often recovered via chemical means. The intended product and any byproduct may be easily and quickly recovered from the reaction mixture thanks to heterogeneous catalysts. This type of catalyst eliminates the need for a washing step. The esterification method using nanocatalysts was proposed to have several benefits, including speedier mixing of the reactants and catalyst and easy and rapid separation from the reaction mixture (Pandit et al. 2023).

Synthesis methods: The methods used to synthesize nanocatalysts may evolve, affecting their structure and properties. Recent advancements might include greener synthesis approaches or techniques that enhance the reproducibility of catalysts.

In addition to the aspects mentioned, the surface chemistry of nanocatalysts can also vary across generations, affecting their catalytic behavior during biodiesel production. The surface chemistry of nanocatalysts can be modified through various methods, such as surface functionalization, doping, or coating, to tune their catalytic activity, selectivity, and stability. For instance, surface functionalization with organic molecules or inorganic ions can enhance the catalyst’s selectivity for specific reactions or improve its compatibility with the reaction medium. The use of nanocatalysts in biodiesel production also presents some challenges, such as the aggregation, fouling, and leaching of active species. These issues can lead to a decrease in catalytic activity and selectivity, as well as an increase in production costs. To address these challenges, researchers are exploring various strategies, such as surface modification, stabilization techniques, and immobilization methods, to improve the stability and reusability of nanocatalysts. In summary, the distinct behavior of nanocatalysts during biodiesel production is influenced by various factors, including catalyst type, particle size, functionalization, surface chemistry, synthesis methods, and stability. The optimization of these factors can lead to more efficient, selective, and sustainable biodiesel production processes. However, further research is needed to fully understand the underlying mechanisms and to develop new generations of nanocatalysts with enhanced performance and stability.

## Transesterification reaction mechanism (alcoholysis)

**Fig. 7:**
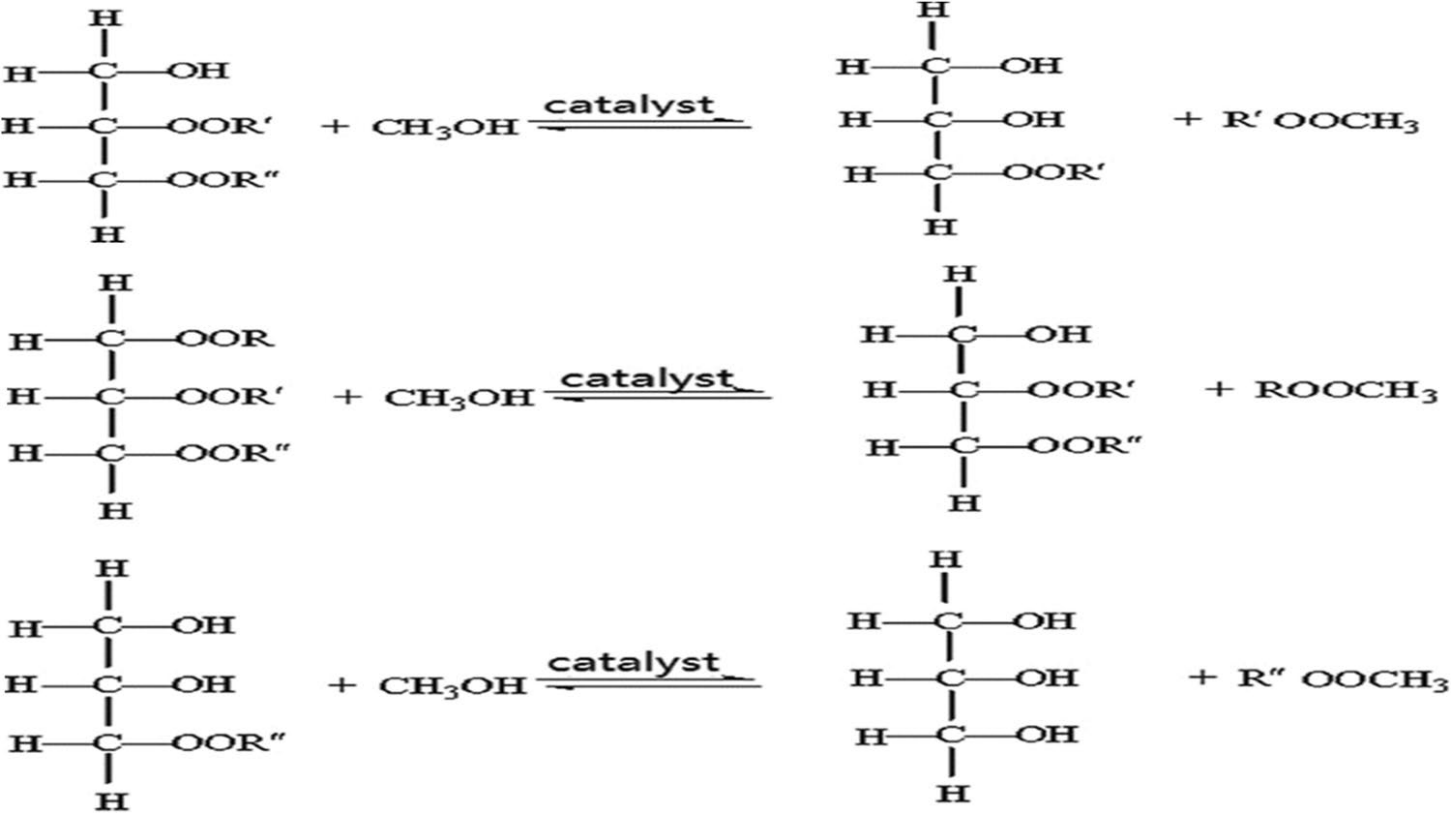
Continuous reversible processes of transesterification reactions

**Fig. 8:**
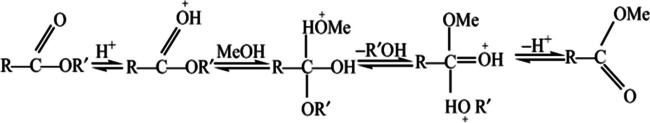
Acid-catalyzed alcoholysis reaction mechanism

**Fig. 9:**
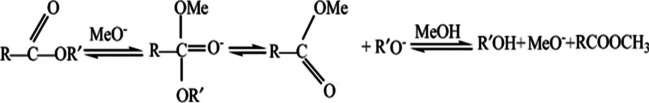
Base-catalyzed alcoholysis reaction mechanism

**Fig. 10:**
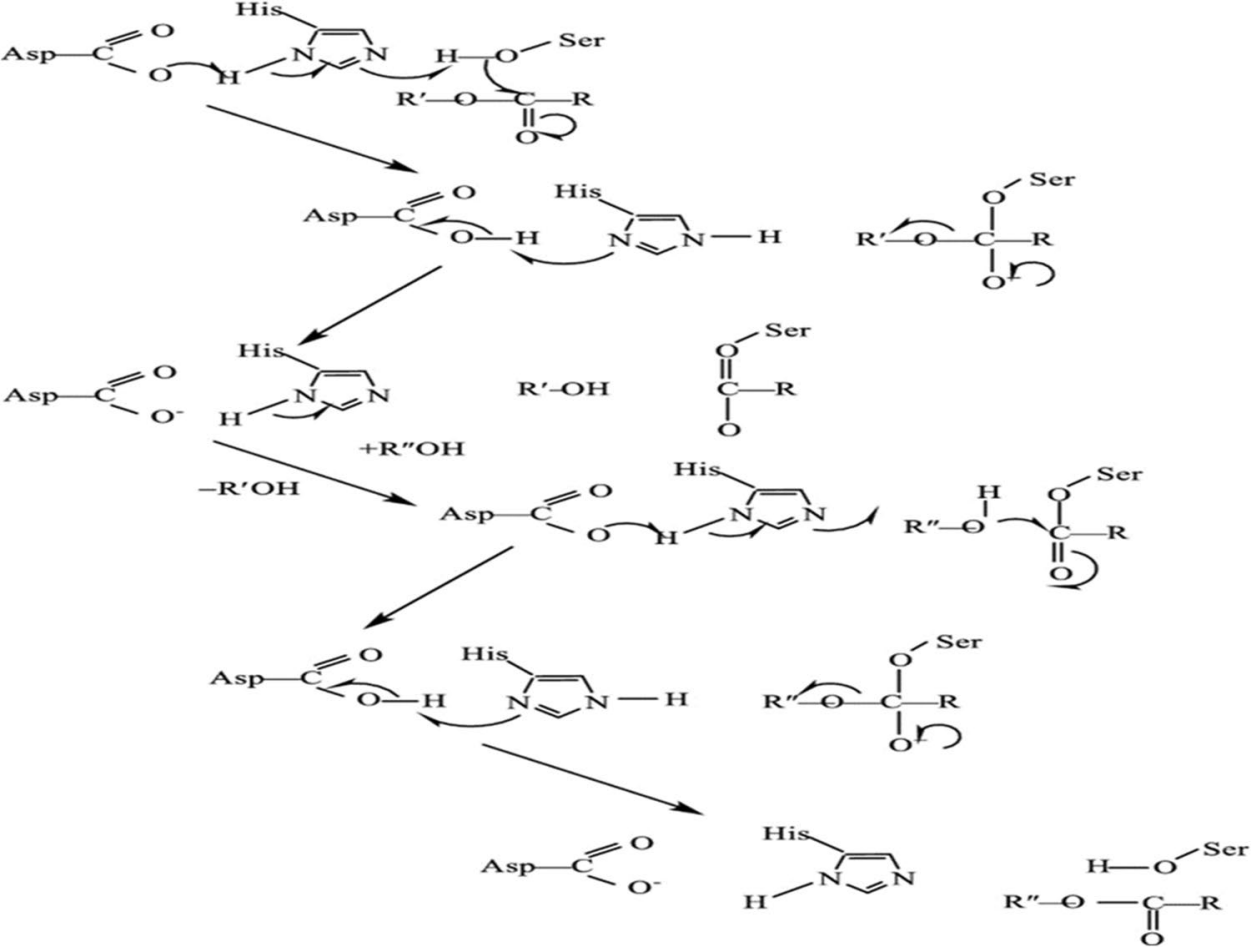
Enzyme-catalyzed alcoholysis reaction mechanism

A large variety of exchange reactions involving oils, fats, and other reactants may be explained by the reaction mechanism. This comprises three processes: (1) transesterification, a rearrangement that yields monoglyceride, diglyceride, or other esters; (2) acidolysis, which involves exchanging fatty acids to produce specific fatty acid products; and (3) alcoholysis, which produces methyl esters in reactions with monohydric alcohols and monyl glycerol in reactions with polyhydric alcohols. Natural vegetable oils, animal fats, and food industry waste oil may all be utilized as source materials for transesterification, a process that produces biodiesel. Methanol, ethanol, propanol, butanol, and pentanol are among the alcohols that can be utilized for transesterification. Because it is a cheap, short-chain, strong polar raw material that reacts rapidly with fatty acid glycerides, methanol is the most widely used of them. Also freely soluble in methanol are base catalysts. A catalytic agent in this reaction might be an acid, base, or enzyme. Base catalysts consist of carbonate, NaOH, KOH, and potassium and sodium alkaloids. Acid catalysts might be hydrochloric, phosphoric, or sulfuric acids. The enzyme lipase is a good catalyst for the esterification of alcohols to fatty acid glycerides. Figures 7, 8, 9, and 10 represent continuous reversible processes for transesterification reactions; every reaction yields a distinct type of alcohol (Kang et al. 2015; Sait et al. 2022; Li et al. 2020; Oyekunle et al. 2023).

## Kinetics

### Kinetic modeling’s significance for process optimization

Kinetic models of chemical processes are powerful tools for reactor design. The kinetic models are very helpful in choosing the best reaction conditions (temperature, pressure, mixing rate, etc.) for chemical or biochemical transformations in reactors or bioreactors. This maximizes the formation of desired products with the least material investment and financial resources. This also holds true for the many techniques used to produce biodiesel, such as homogeneous, heterogeneous, enzyme catalysis, and others. One of the most important stages in the development of chemical processes for industrial applications is thought to be carefully thought-out experimental research and the subsequent creation of a kinetic model (Trejo-Zárraga et al. 2018). Portha et al. (2012) were able to decrease the extra ethanol used in the transesterification reaction in a continuous mode. By adjusting the temperature of the second reactor and adding methanol in stages, they were able to enhance the system’s overall performance, as demonstrated by the results of their simulation. Using triolein as a model chemical, the authors conducted experiments and discovered that it was beneficial to convert diglyceride and monoglyceride in the second reactor and the majority of triolein in the first. Additionally, their calculations suggested that to improve reaction rates at this point, it would be prudent to raise the temperature in the second reactor. Additionally, the authors computed internal concentration profiles using a reactor model that included the kinetic model. They discovered the limiting phenomenon in the overall transformation. To get a deeper comprehension of the rates of output and the inhibitory patterns seen in the transformation scheme, a kinetic model may also be strategically employed (Firdaus et al. 2016). For instance, a reaction scheme for the enzymatic creation of biodiesel might consider many more reaction stages and, consequently, a greater number of parameters. This adds difficulty to the kinetic model creation process, but once this model is solved, it may be utilized to construct an enzyme-catalyzed reactor and eventually optimize the process. The use of kinetic models, which can faithfully replicate the process at various reaction conditions, is helpful in the field of research and process improvement as it offers guidelines for additional experimental work and helps eliminate potentially fruitless experimental trials. Additionally, models may be utilized to foresee how composition will affect the final product’s quality. A model might forecast, for instance, how the feedstock’s water content or FFA may impact the reaction conversion and, in turn, the biodiesel’s production and quality.

### Kinetics of transesterification

Few studies have dealt with kinetic modeling; most of the heterogeneous catalysis research has been on the manufacture and utilization of catalysts. To achieve reaction conditions with inherent kinetics and minimal effects, efforts have been focused on using tiny solid particles. It has been discovered that most heterogeneous transesterifications adhere to a pseudo-first-order model. For instance, Kaur and Ali (2014) discovered that the ethanolysis of *Jatropha curcas L.* oil, which were catalyzed by 15-Zr/CaO-700, adhered to a pseudo-first-order rate law. The Koros-Nowak test proved that the transit impacts were insignificant. Lukić et al. (2014) also discovered a first-order reversible rate law under ideal circumstances for the transesterification of sunflower oil. Table 6 lists some kinetic modeling studies of heterogeneous transesterification.

**Table 6:** List of some kinetic modeling studies of heterogeneous transesterification

Feedstock	Catalyst	Experimental conditions	Kinetic studies	References
		Temperature (°C) M:O molar ratio Mixing speed (rpm)	Kinetic model rate constant (*k*) activation energy (*E*_a_)	
Soybean oil	Amberlyst A_2_6-OH basic ion-exchange resin	50 °C-10:1–550 rpm	Eley–Rideal *k* = 1.94 h.^1^ *k* = 7.48 × 10^−4^ h^−1^	Jamal et al. (2015)
*Jatropha curcas* L	Zr/CaO	65 °C-15:1–500 rpm	Pseudo-first-order *k* = 0.062 min^−1^ *E*_a_ = 29.8 kJ mol^−1^	Kaur and Ali (2014)
Sunflower oil	CaO	60 °C-6:1–900 rpm	Miladinovic model *k* = 0.063 dm^6^mol^−2^ min^−1^	Tasić et al. (2015)
Waste cooking oil	NaOH/chitosan-Fe_3_O_4_	65 °C-6.5:1–500 rpm	Pseudo-first-order *k* = 260.05 min^−1^ *E*_a_ = 21 kJ/mol	Helmi and Hemmati (2021)
Sunflower oil	Ca(OH)_2_	60 °C-6:1–900 rpm	Pseudo-first order *k* = 0.07(1 − exp(− *C*_cat_/2.86); min^−1^	Stamenković et al. (2010)
Used frying oil	NaOH	55 °C-4:1–300 rpm	Pseudo-first-order *k* = 545.65 min^−1^ *E*_a_ = 23.61 kJ/mol	Haryanto et al. (2020)
Sunflower oil	CaO	60 °C-6:1–900 rpm	Pseudo-first order *k* = 0.07 min^−1^	Veljković et al. (2009)
Canola oil	Mg–Co–Al–La HDL	170–200 °C-16:1–900 rpm	First order *E*_a_: 60.5 kJ/mol	Li et al. (2009)
Waste cooking oil	CaO·ZnO 2 wt %	96 °C-10:1–300 rpm	Pseudo-first-order *k* = 0.170 min^−1^	Lukić et al. (2013)
Used cooking oil	Nano-cobalt-doped ZnO	50–80 °C-3:1–136 rpm	Pseudo-second-order *k* = 0.0052 min^−1^	Noreen et al. (2021)
Waste cooking oil	Heteropoly acid, 10 wt %	70 °C-70:1–300 rpm	First order *k* = 0.1062 min^−1^ *E*_a_ = 53.99 kJ/mol	Talebian-Kiakalaieh et al. (2013)

## Characterization methods for the assessment of produced biodiesels

Characterization methods for the assessment of produced biodiesel include various analytical techniques to evaluate the quality and properties of biodiesel. These methods are essential for ensuring that biodiesel meets the required standards and specifications for use as a sustainable and efficient alternative fuel source. The American Society for Testing and Materials (ASTM) is a prominent organization that provides authoritative guidelines for biodiesel testing and characterization methods.

The most common characterization methods for assessing produced biodiesel include the following.

Fatty acid methyl ester (FAME) analysis: FAME analysis is a fundamental method for biodiesel characterization, involving the determination of the fatty acid methyl ester content in biodiesel. This analysis is typically performed using gas chromatography (GC) or high-performance liquid chromatography (HPLC) to quantify individual FAME components, which provides valuable information about the biodiesel’s composition and purity.

Viscosity measurement: Viscosity is a crucial parameter for biodiesel quality assessment, as it affects the flow behavior and performance of the fuel. Dynamic viscosity measurements are commonly conducted to determine the resistance of biodiesel to flow under specific conditions, offering insights into its suitability for use in engines and transportation applications.

Oxidation stability testing: Biodiesel’s resistance to oxidation is an important characteristic that influences its shelf life and storage stability. Various methods, such as the Rancimat test and the PetroOXY test, are employed to assess the oxidation stability of biodiesel by measuring its susceptibility to oxidative degradation over time.

Cold flow properties analysis: The cold flow properties of biodiesel, including cloud point and pour point, are critical factors affecting its performance in cold weather conditions. Characterization methods such as differential scanning calorimetry (DSC) and automated cloud and pour point analyzers are utilized to determine these properties, ensuring that biodiesel remains operational at low temperatures.

Acid value determination: The acid value of biodiesel indicates its acidity level, which can impact engine components and fuel system integrity. Acid value determination involves titration methods to quantify the amount of free fatty acids present in biodiesel, enabling the assessment of its corrosiveness and potential impact on engine performance.

Calorific value measurement: Calorific value, also known as heating value, represents the energy content of biodiesel and is crucial for evaluating its combustion efficiency and heat output. Bomb calorimetry is commonly used to measure the calorific value of biodiesel, providing essential data for assessing its energy potential as a fuel source.

Sulfur content analysis: Sulfur content determination is essential for ensuring compliance with environmental regulations and assessing the environmental impact of biodiesel combustion. Techniques such as X-ray fluorescence (XRF) spectroscopy or ultraviolet fluorescence analysis are employed to measure sulfur levels in biodiesel samples.

Glycerol content quantification: Glycerol content in biodiesel must be monitored to ensure compliance with quality standards and prevent potential issues related to fuel stability and engine performance. Analytical methods like gas chromatography coupled with flame ionization detection (GC-FID) are utilized for the accurate quantification of glycerol in biodiesel products.

These characterization methods collectively provide comprehensive insights into the chemical composition, physical properties, stability, and environmental impact of produced biodiesel, supporting quality control measures and regulatory compliance within the biofuel industry.

## The recent development in biodiesel production

Biodiesel, a renewable and sustainable alternative to conventional diesel fuel, has seen significant developments in recent years. These advancements have focused on improving the efficiency of biodiesel production processes, expanding feedstock options, and enhancing the overall sustainability of biodiesel as a viable energy source. One notable recent development is the use of advanced catalysts in biodiesel production. Catalysts play a crucial role in the conversion of vegetable oils or animal fats into biodiesel through a process called transesterification. Researchers have been exploring various catalysts, such as solid acid catalysts, enzyme catalysts, and heterogeneous catalysts, to improve reaction rates, reduce energy consumption, and enhance biodiesel quality. These catalysts offer advantages like higher conversion rates, milder reaction conditions, and easier separation of the catalyst from the product (Garcia-Silvera et al. 2023). Another significant development is the utilization of non-traditional feedstocks for biodiesel production. While conventional biodiesel feedstocks include soybean oil and rapeseed oil, researchers have been investigating alternative sources such as algae, waste cooking oil, and non-food crops like jatropha and camelina. Algae have gained attention due to their high oil content and ability to grow in various environments. The use of non-traditional feedstocks helps to reduce competition with food production and enhances the overall sustainability of biodiesel (Garg et al. 2023). Furthermore, efforts have been made to improve the sustainability of biodiesel production by reducing its environmental impact. This includes optimizing production processes to minimize water and energy consumption, reducing greenhouse gas emissions, and implementing waste management strategies. Additionally, researchers have been exploring the concept of “second-generation” biodiesel, which involves utilizing waste materials, such as agricultural residues and lignocellulosic biomass, to produce biodiesel. This approach not only reduces waste but also maximizes resource utilization (Makepa et al. 2023).

## Biodiesel engine performance and emissions

Compared to petrodiesel fuel, burning biodiesel releases fewer particulates, carbon monoxide, and unburned hydrocarbons. Since biodiesel is produced using natural resources, its sulfur content is relatively low, which means that when it burns in an engine, it releases less sulfur dioxide into the atmosphere (Rayati et al. 2020). All biodiesels and their blends have shown the capacity to enhance gas turbine performance while lowering emissions of carbon dioxide, carbon monoxide, nitrogen oxide, and hydrocarbons under a range of operating conditions. To employ fuels in an engine, one must be aware of their characteristics for combustion. Although fossil fuel-based diesel fuel may not be entirely replaced by biodiesel, it can aid in achieving balanced energy utilization. One benefit is that biodiesel may be used in contemporary engines with little modification. Older vehicles with natural rubber gasoline lines, however, require a few modifications. Rubber fuel lines must be replaced since they will crack when used with biodiesel. On the other hand, an oil or gasoline dilution in the fuel system is possible in a modern vehicle with a DPF (diesel particulate filter). The ability of gasoline to lubricate the fuel injection system is believed to be crucial for diesel engines. The use of diesel–biodiesel mixes can thereby enhance their general lubricity. Additionally, the lower sulfur level of today’s diesel fuel could affect its lubricity because the compounds that provided lubrication are no longer present (Veza et al. 2022).

## Techno-economic analysis

Techno-economic analysis (TEA) plays a crucial role in assessing the economic feasibility and viability of biodiesel production processes. It involves evaluating the overall costs, revenues, and profitability of biodiesel production, considering various factors such as feedstock costs, capital investment, operational expenses, and market prices. Recent studies have employed TEA to analyze and optimize biodiesel production processes, providing valuable insights for decision-making and process design. One example of TEA in biodiesel production is a study conducted by Zhang (2021), which evaluated the techno-economic performance of different feedstocks and process configurations for biodiesel production. The analysis considered factors such as feedstock availability, conversion efficiency, capital costs, operating costs, and market prices. The study highlighted the importance of feedstock selection and process optimization in achieving cost-effective biodiesel production. Another study by Tasić (2020) performed TEA for manufacturing biodiesel from used cooking oil. The analysis included the estimation of capital and operational costs, energy consumption, and environmental impacts. The study demonstrated the economic feasibility of waste cooking oil-based biodiesel production and identified critical parameters affecting the overall economics of the process. Furthermore, a study by Atabani (2020) conducted TEA for biodiesel production from microalgae. The analysis considered various scenarios, including different cultivation systems and conversion technologies. The study assessed the economic viability of microalgae-based biodiesel production, considering factors such as biomass productivity, lipid content, capital investment, and operational costs. These recent studies emphasize the importance of TEA in evaluating the economic aspects of biodiesel production. By considering a comprehensive range of factors, TEA provides valuable insights into the cost-effectiveness, profitability, and sustainability of biodiesel production processes, helping guide decision-making and process optimization.

## Challenges, perspectives, and further research

The homogeneous catalyst has been thoroughly examined, and the literature has addressed several issues. However, heterogeneous catalysts are a very new field of study, and there is now a lot of research being done in this area. The literature has documented many obstacles regarding these catalysts:

- It has been said that the primary issues with heterogeneous catalysts are instability, reduced reaction rate, and short catalyst life.
- It has been reported that solid-base catalysts are FFA, CO_2_, and water sensitive. Through saponification, they destroy the catalyst and render it inactive.
- Because water hydrolyzes the ionic group in solid acid catalysts, leaching and product contamination have been documented.
- It has been documented that during enzymatic transesterification, methanol causes lipase inhibition.
- In the case of nanocatalysts, good performance requires increasing the reaction time under very moderate working conditions. To attain normal reaction times, however, harsh working conditions must be used, which raises energy consumption.

Future research should pay attention to the following recommendations:

- To create novel catalysts with enhanced catalytic performance, more research into waste-derived catalysts is required.
- The creation of highly selective and active heterogeneous catalysts that may be used in industrial settings at a reasonable cost.
- Investigating novel catalyst supports with a network of linked pores of the right size and a selected surface area.
- Investigating the use of waste or biomass as a catalyst source to lower related costs and increase sustainability for solid catalysts that are sold commercially.
- Improving synthesis catalyst production procedures and treatment stages to move the technology from a lab to an industrial setting.
- Maintaining the high basic strength of the synthesis catalyst while improving the shape and sensitivity to FFA and water.
- Additional research is needed into the manufacturing of industrial enzymatic biodiesel as a guaranteed choice for the future.
- To give relevant information about the suitable catalyst, future research on the catalytic mechanism of partial catalysts should examine the features in depth.
- Ways to recover and reuse nanocatalysts that are both economical and energy efficient.
- Kinetic studies of the biodiesel production reaction using the synthesized catalyst should be carried out.

## Conclusion

This extensive review delves into the various aspects of biodiesel production and its promise as a sustainable alternative for a greener energy future. The significance of feedstock selection and preparation is emphasized, with effective techniques discussed for optimizing biodiesel production efficiency and quality. Biodiesel has emerged as a versatile and promising alternative for transportation, industrial processes, and energy generation, demonstrating its potential to reduce greenhouse gas emissions and dependency on fossil fuels. The key process of transesterification is thoroughly examined, encompassing the utilization of diverse catalysts, including homogeneous, heterogeneous, enzyme based, and nanomaterials. The unique characteristics and performance of nanomaterials in transesterification are highlighted, offering prospects for enhanced efficiency and selectivity. Understanding the reaction mechanism and kinetics of transesterification is crucial for optimizing the production process. Kinetic modeling is identified as a valuable tool for process optimization, enabling better control and improved production efficiency. Methods for assessing the quality and properties of produced biodiesel are discussed, highlighting the importance of accurate characterization to meet quality standards and ensure compatibility with engine systems. Recent developments in biodiesel production showcase progress in feedstock selection, process optimization, and sustainability. However, challenges related to engine performance, emissions, and compatibility remain obstacles to wider biodiesel adoption. Future research should focus on addressing these challenges through innovative engine technologies, improved fuel formulations, and effective emission control strategies. Techno-economic analysis provides insights into the economic feasibility of biodiesel production, considering factors such as feedstock costs, process efficiency, and market demand. Ongoing analysis and assessment are essential for ensuring the commercial viability and scalability of biodiesel production. In conclusion, biodiesel presents a promising sustainable solution, but its advancement requires continuous research, development, and collaboration among academia, industry, and policymakers. Addressing challenges, pursuing further research, and implementing the recommendations outlined in this review will contribute to the widespread adoption of biodiesel as a renewable energy source, paving the way for a cleaner and more sustainable future.

## Data Availability

Data available on request from the authors.
